# Epigenetic regulation in spinal muscular atrophy: emerging areas and future directions

**DOI:** 10.1186/s13023-025-03857-3

**Published:** 2025-07-10

**Authors:** Haoran Li, Bo Yu, Ye yuan, Nannan Chen, Jimeng Wu, Zhiqing Zhang

**Affiliations:** 1 Department of Pharmacy, The Second Hospital of Hebei Medical University, 215 Heping West Road, Shijiazhuang, 050000 Hebei Province China; 2 Department of Pediatrics, The Second Hospital of Hebei Medical University, Shijiazhuang, 050000 China; 3 Department of Pharmacy, The Second Hospital of Hebei Medical University, Shijiazhuang, 050017 China; 4 School of Basic Medicine, Chengde Medical College, Chengde, 067000 China

**Keywords:** Spinal muscular atrophy, Epigenetics, Environmental factors, *SMN2* gene

## Abstract

Spinal Muscular Atrophy (SMA) is a neuromuscular disorder precipitated by mutations or deletions in the Survival Motor Neuron 1 (*SMN1*) gene. Although the *SMN2* gene partially compensates for SMN1 functional deficiency, its expression is regulated by complex epigenetic and environmental factors. This review comprehensively elucidates the regulatory mechanisms through which epigenetic modifications-encompassing DNA methylation, histone modifications, and non-coding RNAs-modulate *SMN2* gene expression and impact SMA pathogenesis and progression. We also briefly discuss how these epigenetic mechanisms may interact with selected environmental factors in modifying disease outcomes. Emerging evidence suggests that these epigenetic factors and environmental exposures interact synergistically to influence disease trajectory and may account for the heterogeneity observed in SMA clinical manifestations. These insights have given rise to novel therapeutic strategies, including pharmacological interventions targeting epigenetic pathways and optimized management of environmental factors. Integrating multi-omics analyses holds promise for advancing personalized precision medicine approaches for SMA and potentially improving patient outcomes.

## Introduction

Spinal Muscular Atrophy (SMA) is a severe neuromuscular disorder characterized by the progressive degeneration of motor neurons, resulting in progressive muscle weakness and atrophy [1]. As a rare autosomal recessive genetic disease, SMA exhibits an incidence rate of approximately 1/6,000–1/10,000 live births [2]. The primary pathogenic gene associated with SMA is the Survival Motor Neuron 1 (*SMN1*) gene, located in the 5q13 region of the human chromosome. This gene encodes the SMN protein, which plays a crucial role in motor neuron survival and function [3]. Most SMA patients exhibit homozygous deletions or other loss-of-function mutations in the *SMN1* gene, resulting in significantly diminished levels of functional SMN protein [4]. The human genome contains *SMN2*, a highly homologous gene to *SMN1*, differing by only five nucleotides in its coding sequence. The silent C > T mutation in exon 7 of SMN2 causes alternative splicing during transcription, yielding only approximately 10% of full-length functional SMN protein [5]. The copy number of SMN2 represents one of the principal determinants of the severity of the SMA clinical phenotype, with higher copy numbers correlating with increased production of functional SMN protein and generally milder clinical manifestations [6].

Although the primary genetic etiology of SMA has been established, the heterogeneity in phenotypic severity and disease progression among patients suggests the involvement of additional factors beyond gene deletions and mutations in the pathogenic process and disease progression [7]. This clinical heterogeneity has directed research attention toward epigenetic regulation as a critical modulator of SMA pathogenesis and progression. Epigenetic modifications represent heritable changes that influence gene expression without altering the underlying DNA sequence, encompassing sophisticated regulatory mechanisms such as DNA methylation, histone modifications, chromatin remodeling, and non-coding RNA regulation [8, 9]. These epigenetic processes have emerged as fundamental determinants of *SMN2* gene expression levels, thereby regulating the production of functional SMN protein and ultimately influencing disease severity and progression [10].

Recent advances in epigenetic research have begun to reveal that SMA pathogenesis may involve complex multilayered regulatory networks that extend beyond the *SMN1* gene deletion paradigm. Emerging studies suggest that DNA methylation patterns, particularly in the *SMN2* promoter region, may correlate with disease severity, while histone modifications appear to create dynamic chromatin landscapes that potentially influence *SMN2* transcriptional accessibility [11, 12]. Additionally, non-coding RNAs, including microRNAs and long non-coding RNAs, are increasingly recognized as contributors to post-transcriptional regulatory circuits that may fine-tune SMN protein expression [13]. Understanding how these epigenetic regulatory mechanisms interact and whether they form integrated networks that collectively influence the cellular response to *SMN1* deficiency represents an important area of ongoing investigation.

The clinical significance of epigenetic regulation in SMA extends beyond mechanistic understanding to therapeutic implications. Unlike genetic mutations, epigenetic modifications are potentially reversible, offering new opportunities for therapeutic intervention. This reversibility has led to the development of epigenetic-targeted therapies, including DNA methyltransferase inhibitors, histone deacetylase inhibitors such as valproic acid and trichostatin A, and antisense oligonucleotides targeting regulatory RNAs, which have shown therapeutic effects in preclinical and clinical studies [14–16]. Additionally, emerging evidence suggests that certain environmental factors may influence SMA progression through epigenetic mechanisms, creating a complex interplay between genetic predisposition, epigenetic regulation, and environmental modulation [17, 18]. Understanding these gene-environment-epigenome interactions may reveal additional therapeutic targets and inform personalized treatment strategies [9].

The investigation of epigenetic regulatory mechanisms in SMA also contributes to broader insights into neurodegenerative diseases and motor neuron biology. Emerging evidence suggests that epigenetic dysfunction may represent a common pathological feature across motor neuron diseases, indicating the potential importance of chromatin regulation in neuronal survival and function [19]. Furthermore, research into epigenetic modifications as potential disease biomarkers may provide new approaches for disease monitoring, prognosis assessment, and therapeutic response evaluation.

This review aims to systematically synthesize and analyze recent advances in SMA epigenetic research. It examines how epigenetic modifications influence disease onset, progression, and therapeutic outcomes, while also exploring how environmental factors may interact with these epigenetic processes. By integrating multifaceted research findings, we hope to provide insights for basic research and clinical practice in SMA management and to identify current research challenges and future directions.

## Epigenetic studies of SMA

Although *SMN1* gene deletion represents the primary genetic cause of SMA, the clinical heterogeneity observed among patients with identical genetic backgrounds has highlighted the critical importance of epigenetic regulatory mechanisms in disease manifestation and progression. Comprehensive molecular studies have demonstrated that epigenetic modifications serve as key determinants of *SMN2* gene expression efficiency, thereby influencing the compensatory SMN protein production that ultimately shapes disease severity. These regulatory mechanisms operate through multiple interconnected molecular pathways that collectively control *SMN2* transcriptional activity and contribute to the complex networks governing SMA pathogenesis.

### Epigenetic regulation of the *SMN2* gene

**Fig. 1:**
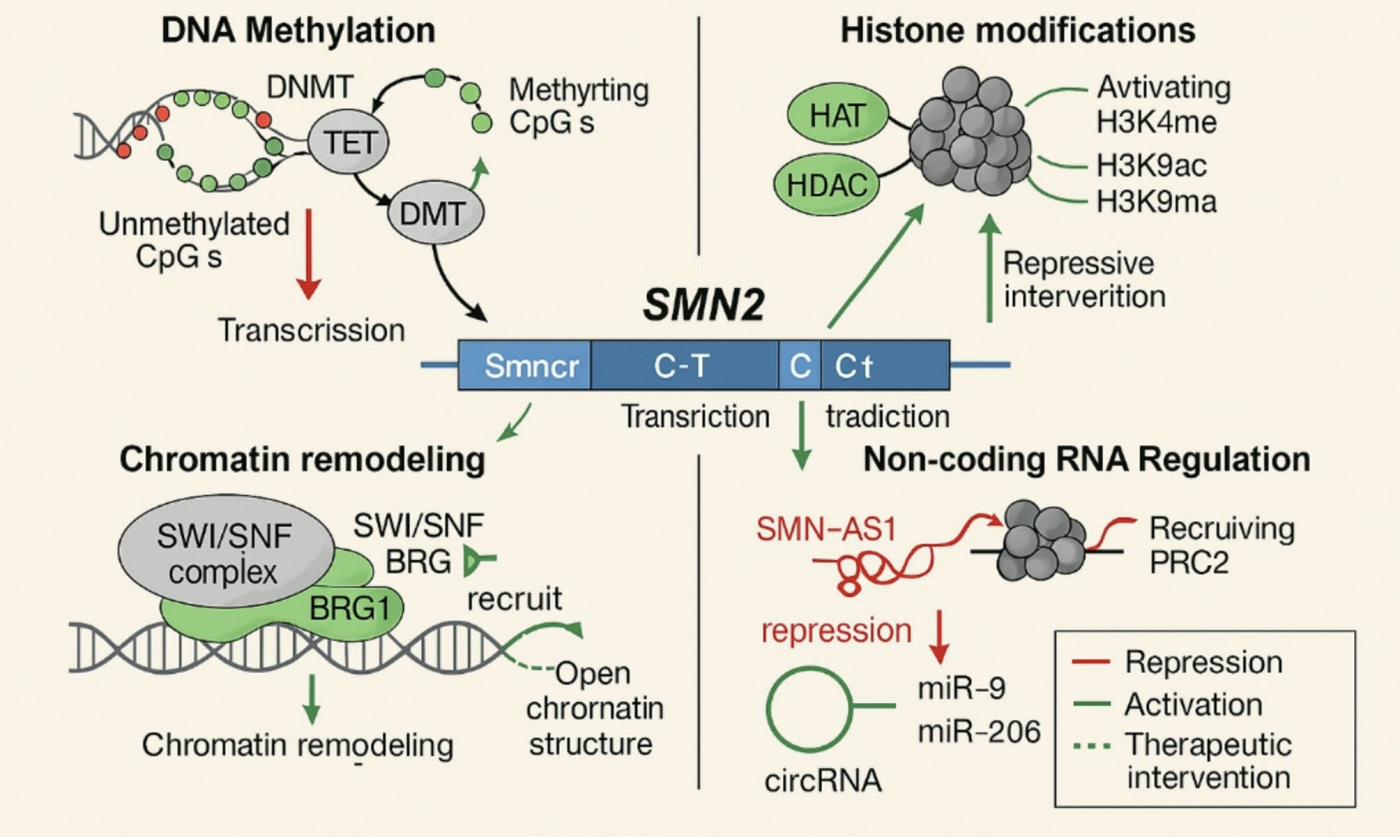
Epigenetic regulatory mechanisms controlling *SMN2* gene expression in spinal muscular atrophy Notes: Four major epigenetic pathways influencing *SMN2* expression: DNA methylation (top left), histone modifications (top right), chromatin remodeling (bottom left), and non-coding RNA regulation (bottom right). Green arrows indicate activation; red arrows indicate repression of gene expression

The epigenetic regulation of the *SMN2* gene plays an important role in SMA pathophysiology. Despite only five nucleotide differences in coding sequences between *SMN2* and *SMN1*, a silent mutation in exon 7 of *SMN2* results in the skipping of this exon during splicing in most transcripts, resulting in a functionally insufficient SMN protein [20]. Consequently, the *SMN2* gene represents a critical therapeutic target in SMA, as increasing *SMN2* expression could potentially compensate for the protein deficiency resulting from the *SMN1* gene deletion. Multiple studies have demonstrated that *SMN2* gene expression is subject to complex epigenetic regulation, with regulatory mechanisms primarily involving epigenetic modifications across various regions of the *SMN2* gene. The interplay of these diverse epigenetic mechanisms in controlling *SMN2* expression is illustrated in Fig. 1.

#### Epigenetic modifications of the promoter region

The levels of DNA methylation and histone acetylation in the promoter region of the *SMN2* gene exhibit an inverse correlation with gene expression. Studies have found that antisense oligonucleotides targeting the *SMN2* promoter region can increase the expression of *SMN2* in SMA cell lines and mouse models [21]. This suggests that modulation of the epigenetic state of the promoter region can affect *SMN2* transcription levels. Demethylating agents and histone deacetylase (HDAC) inhibitors represent two primary classes of epigenetic regulatory drugs employed to augment *SMN2* expression. The study showed that combined administration of the HDAC inhibitor LBH589 and splice-switching antisense oligonucleotides significantly increased the expression of full-length functional SMN protein from *SMN2* in SMA cells [16]. This result underscores the importance of histone acetylation in regulating *SMN2* expression.

#### Epigenetic modifications of intronic regions

Besides the promoter region, the intronic sequences of the *SMN2* gene play a crucial role in regulating gene expression. These intronic regions contain multiple cis-regulatory elements that regulate the formation of mature mRNA by affecting pre-mRNA splicing. Investigations have identified an intronic splicing silencer (ISS) within intron 7 of the *SMN2* gene that inhibits the insertion of exon 7, resulting in the production of unstable delta-7 (Δ7) SMN protein [22]. The presence of ISS, leading to the production of unstable Δ7 isoforms, represents a significant factor contributing to the insufficient production of functional SMN protein from *SMN2*. Further studies have revealed additional intronic elements affecting *SMN2* splicing. Notably, genetic variation in intron 6 significantly impacts *SMN2* splicing efficiency, with the G allele at the A-44G polymorphic site enhancing recognition of exon 7 [23]. This intronic variation may exert its effects through alterations in RNA secondary structure or by influencing splicing factor binding. Recently discovered splicing inhibitors have been shown to prevent the inclusion of exon 7 by recruiting inhibitors such as *RALY* and hnRNP C [24]. SMN protein deficiency affects its gene splicing and leads to genome-wide intron retention and DNA damage, suggesting a crucial role for SMN in maintaining overall genome stability [25]. The studies have further elucidated this mechanistic pathway, revealing that SMN deficiency disrupts spliceosome assembly, resulting in aberrant splicing of specific DNA repair genes [26]. Additionally, experimental evidence has confirmed the critical role of SMN in maintaining R-loop homeostasis, and its deficiency leads to abnormal transcription and splicing of essential neuronal genes [27]. These findings are important for developing therapeutic strategies targeting *SMN2* splicing regulation [28].

#### Chromatin regulation of *SMN2* gene expression

*SMN2* gene expression is regulated by local epigenetic modifications and higher-order chromatin structure and nuclear environment. The open or closed state of chromatin determines the accessibility of transcription factors and regulatory elements to DNA, affecting gene transcription activity. Studies have demonstrated that the chromatin state of the *SMN2* locus closely correlates with its transcriptional levels. Active histone modifications such as H3K9 acetylation and H3K4 trimethylation are associated with increased *SMN2* expression, while repressive marks like H3K9 methylation are associated with *SMN2* silencing [29]. This suggests that chromatin epigenetic reprogramming is crucial in regulating *SMN2* expression. The combined use of splicing correction antisense oligonucleotides (ASOs) and chromatin regulators such as HDAC inhibitors can enhance the therapeutic effect, highlighting the importance of integrating multiple epigenetic regulatory strategies [30]. In the chromatin environment, SMN protein deficiency leads to abnormal accumulation of R-loop structures, subsequently inducing DNA damage and genomic instability [31]. Concurrently, the zinc finger protein *ZPR1* binds to the *SMN2* locus and recruit’s chromatin remodeling factors, promoting R-loop dissolution and *SMN2* transcription.

### The role of DNA methylation in SMA

DNA methylation represents a focal point in epigenetic research and a critical entry point for understanding SMA pathogenesis and therapeutic development. Increasing evidence shows that SMA patients have widespread DNA methylation abnormalities, which are not limited to the *SMN2* gene but involve multiple genes related to the occurrence and development of the disease throughout the genome.

#### Abnormal methylation of the *SMN2* promoter region

Clinical investigations have revealed significantly elevated methylation levels in the *SMN2* gene promoter region of SMA patients compared to healthy controls, with higher promoter methylation positively correlating with clinical severity [18, 32]. This phenomenon has been validated across diverse populations, suggesting that promoter region methylation status may represent a crucial factor influencing SMA phenotypic variation. Although the exact mechanism by which hypermethylation of the *SMN2* promoter exacerbates the disease phenotype remains fully elucidated, it may involve methylation-mediated inhibition of promoter activity, leading to a decrease in *SMN2* transcription levels. Notably, histone deacetylase (HDAC) inhibitors can partially reverse the hypermethylated state of the *SMN2* promoter, indicating potential interactions between different epigenetic modifications [32].

#### Genome-wide methylation abnormalities

The genome-wide methylation profile of SMA patients showed extensive abnormalities in addition to the *SMN2* gene. High-throughput methylation analyses have revealed hundreds of differentially methylated CpG sites in peripheral blood samples from SMA patients [17]. These abnormally methylated genes are involved in multiple processes closely related to motor neuron function, including neural development, axon guidance, and cell adhesion. Among them, the methylation levels of specific genes such as *SLC23A2* and *NCOR2* were significantly correlated with clinical severity, suggesting that they may be involved in the onset and progression of the disease. Similar methylation abnormalities have been observed in SMA animal models and other neurodegenerative diseases. For instance, the *SOD1* transgenic ALS mouse models exhibit extensive abnormal DNA and RNA methylation in the spinal cord and skeletal muscle tissues [19], indicating that epigenetic aberrations may represent a common molecular signature across motor neuron diseases. This perspective is supported by several studies, especially those studying the molecular mechanism of muscle degenerative changes. DNA methylation regulation has emerged as a focal point of research [10].

#### Therapeutic strategies targeting DNA methylation

Given the pivotal role of DNA methylation abnormalities in the pathogenesis of SMA, therapeutic strategies targeting this mechanism have shown preliminary progress. DNA methyltransferase (*DNMT*) inhibitors, such as 5-azacytidine, have demonstrated promising results in vitro and animal studies, enhancing *SMN2* gene expression and improving motor function in SMA models [33]. However, the clinical application of these agents may face challenges related to off-target effects and toxicity, which require further optimization and evaluation. In addition to direct modulation of DNA methylation, recent studies have revealed the importance of post-translational modifications of the SMN protein, including phosphorylation and ubiquitination, in complex assembly and functional regulation [34, 35]. This provides another possible approach to the development of new treatments. Future research should integrate epigenomic and transcriptomic data to systematically elucidate SMA-specific gene regulatory networks [36, 37].

### Regulation of SMN expression by histone modification and chromatin remodeling

Dynamic changes in histone modifications and chromatin structure represent crucial mechanisms for gene expression regulation and play pivotal roles in the pathogenesis of SMA. Increasing evidence suggests multiple histone modifications and chromatin remodeling factors complexly regulate *SMN2* gene expression.

#### Histone acetylation and smn2 gene expression

Histone acetylation, particularly of H3 and H4, strongly correlates with transcriptional activity. In the *SMN2* gene, the histone acetylation levels in the promoter and exon 7 regions directly influence the expression intensity [38]. This recognition has focused on HDAC inhibitors in SMA therapeutic research. Several HDAC inhibitors, including valproic acid, butyrate, and Trichostatin A, have demonstrated significant upregulation of *SMN2* expression in preclinical and clinical studies [15, 39, 40]. These findings further validate the central role of histone acetylation in regulating *SMN2* gene expression. However, the therapeutic responses to HDAC inhibitors exhibit individual variation, correlating closely with dosage, treatment duration, and cell type specificity. For instance, in SMA patient fibroblasts, SAHA and Dacinostat showed distinct regulatory characteristics at the transcriptional and methylation levels [41]. These observations suggest that developing histone acetylation-based therapeutic strategies requires more precise and personalized dosing regimens.

#### Histone methylation and *SMN2* alternative splicing

Histone methylation is another crucial epigenetic mechanism for fine-tuning *SMN2* expression. The exon 7 region of the *SMN2* gene exhibits a characteristic histone methylation pattern, with significant enrichment of the transcriptional activation mark H3K4 trimethylation and relatively low levels of the repressive mark H3K9 methylation. Functional studies indicate this methylation pattern is closely associated with *SMN2* splicing regulation. Downregulation of H3K4 methyltransferase *SET7/9* activity leads to exon 7 skipping, consequently reducing full-length *SMN2* transcript production [42]. Conversely, inhibition of H3K9 methyltransferase *G9a* promoted exon 7 insertions, enhancing SMN protein expression [40]. These findings reveal precise mechanistic roles for H3K4 and H3K9 methylation regulating *SMN2* alternative splicing. Notably, the SMN protein is not only subject to regulation by histone modification, but also exhibits the characteristics of a chromatin binding protein and can specifically recognize H3K79 demethylation modification [43]. This finding suggests that SMN may perform feedback regulation on chromatin status in gene expression control, although the detailed molecular mechanism and biological significance require further elucidation.

#### Chromatin remodeling complexes and *SMN2* transcription

Chromatin remodeling represents one of the fundamental epigenetic mechanisms for gene expression regulation. The chromatin structure is altered by dynamically regulating the assembly and positioning of nucleosomes, thereby affecting the accessibility of transcription factors and RNA polymerases to gene regulatory sequences. The SWI/SNF chromatin remodeling complex plays a core role in the transcriptional regulation of the *SMN2* gene. Molecular mechanistic studies have revealed that *BRG1*, a key functional component of the SWI/SNF complex, is essential for maintaining open chromatin states at the *SMN2* promoter region. Decreased *BRG1* expression levels significantly reduce chromatin accessibility at the *SMN2* promoter region, impeding RNA polymerase II recruitment and ultimately suppressing transcription initiation, while upregulation of *BRG1* enhances SMN2 transcriptional activity [42]. These findings demonstrate that precise coordination between chromatin remodeling complexes and transcriptional machinery represents an important regulatory mechanism to ensure appropriate *SMN2* gene expression levels.

### Potential roles of non-coding RNAs in SMA pathology

Non-coding RNAs (ncRNAs), crucial regulatory molecules that do not encode proteins, play pivotal roles in gene expression regulatory networks. Advancing research has increasingly emphasized the importance of ncRNAs, especially long non-coding RNAs (lncRNAs), microRNAs (miRNAs), and circular RNAs (circRNAs) in the pathogenesis of SMA, offering novel perspectives for therapeutic strategy development.

#### Long non-coding RNAs

LncRNA is a class of functional RNA molecules with a length of more than 200 nucleotides, which participate in the fine regulation of gene expression through multiple mechanisms. At the SMN gene locus, a crucial antisense lncRNA, *SMN-AS1*, has been identified, whose mechanism of action involves recruiting polycomb repressive complex 2 (*PRC2*) to suppress *SMN2* gene expression. Detailed functional studies have shown that specific inhibition of *SMN-AS1* effectively blocks PRC2 recruitment, thereby relieving *SMN2* expression suppression [44]. The researcher designed an ASOs targeting *SMN-AS1*, which significantly enhance *SMN2* transcription levels and SMN protein synthesis and demonstrate therapeutic efficacy in improving the motor function of SMA mouse models [14]. These results reveal the key role of lncRNA-mediated epigenetic silencing mechanisms in SMA pathogenesis and provide an important theoretical foundation for developing RNA-targeted therapeutic strategies.

#### MicroRNAs

miRNAs are regulatory RNA molecules of approximately 22 nucleotides, primarily inhibiting protein translation by pairing with target gene mRNA.

Systematic transcriptome analyses revealed significant alterations in miRNA expression profiles in SMA patients and disease models, which are closely associated with disease progression. At the molecular level, certain miRNAs exhibit disease-promoting characteristics, such as the upregulation of *miR-9* and *miR-206* in the spinal cord and skeletal muscle of SMA mouse models, accelerating motor neuron death and muscle atrophy through suppression of neurotrophic factor signaling pathways [45]. In contrast, reduced expression levels of neuroprotective miRNAs, such as *miR-431*, *miR-138*, and *miR-146a*, may exacerbate disease phenotypes [13].

#### Circular RNAs

circRNAs, formed by specific back-splicing of precursor mRNA, exhibit high stability and tissue-specific expression patterns due to their unique structure. In the pathological process of SMA, the function of circRNA is gradually being elucidated. A new type of circRNA formed by early exon back-splicing has been identified at the SMN gene locus, capable of interacting with various RNA-binding proteins to regulate gene expression [46]. Through single-cell RNA sequencing technology, researchers mapped the expression profile of circRNAs in the spinal cord tissue of SMA mouse models. They found multiple circRNAs specifically dysregulated in motor neurons [47]. Further investigations have revealed that Cajal body and nucleolar dysfunction in SMA motor neurons may lead to aberrant processing and localization of various nuclear RNAs such as snRNAs and snoRNAs [48].

#### Potential of non-coding RNAs as biomarkers and therapeutic targets for SMA

Noncoding RNA plays a key role in the pathogenesis of SMA and has shown important application value in the domains of disease diagnosis, prognosis assessment, and drug development. Systematic transcriptome analyses have revealed characteristic miRNA expression profile alterations in the cerebrospinal fluid of SMA patients, with molecules such as *miR-132* and *miR-182* emerging as potential diagnostic markers [49]. These findings provide theoretical foundations for developing miRNA-based therapeutic strategies such as ASO or small molecule inhibitors. In addition, circular RNA has unique advantages in biomarker development due to its unique molecular properties, high tissue specificity, and structural stability. The newly discovered circSMN can regulate multiple SMA-related gene expression networks through competitive endogenous RNA mechanisms, providing a new molecular target for therapeutic intervention [46].

In summary, epigenetic regulatory mechanisms play central roles in SMA pathogenesis. Multiple epigenetic mechanisms, including DNA methylation, histone modifications, chromatin remodeling, and non-coding RNAs, regulate *SMN2* gene expression through various pathways. The elucidation of these molecular mechanisms deepens the understanding of the pathological process of SMA and provides an important basis for developing new treatment strategies. However, the transformation from basic research to clinical application faces multiple challenges, such as treatment specificity, safety assessment, and delivery system optimization. Future research directions should focus on integrating multi-omics data analysis to systematically analyze SMA’s molecular network dysregulation mechanisms, thereby establishing foundations for achieving personalized precision treatment.

## Modulatory factors in SMA pathogenesis

**Fig. 2:**
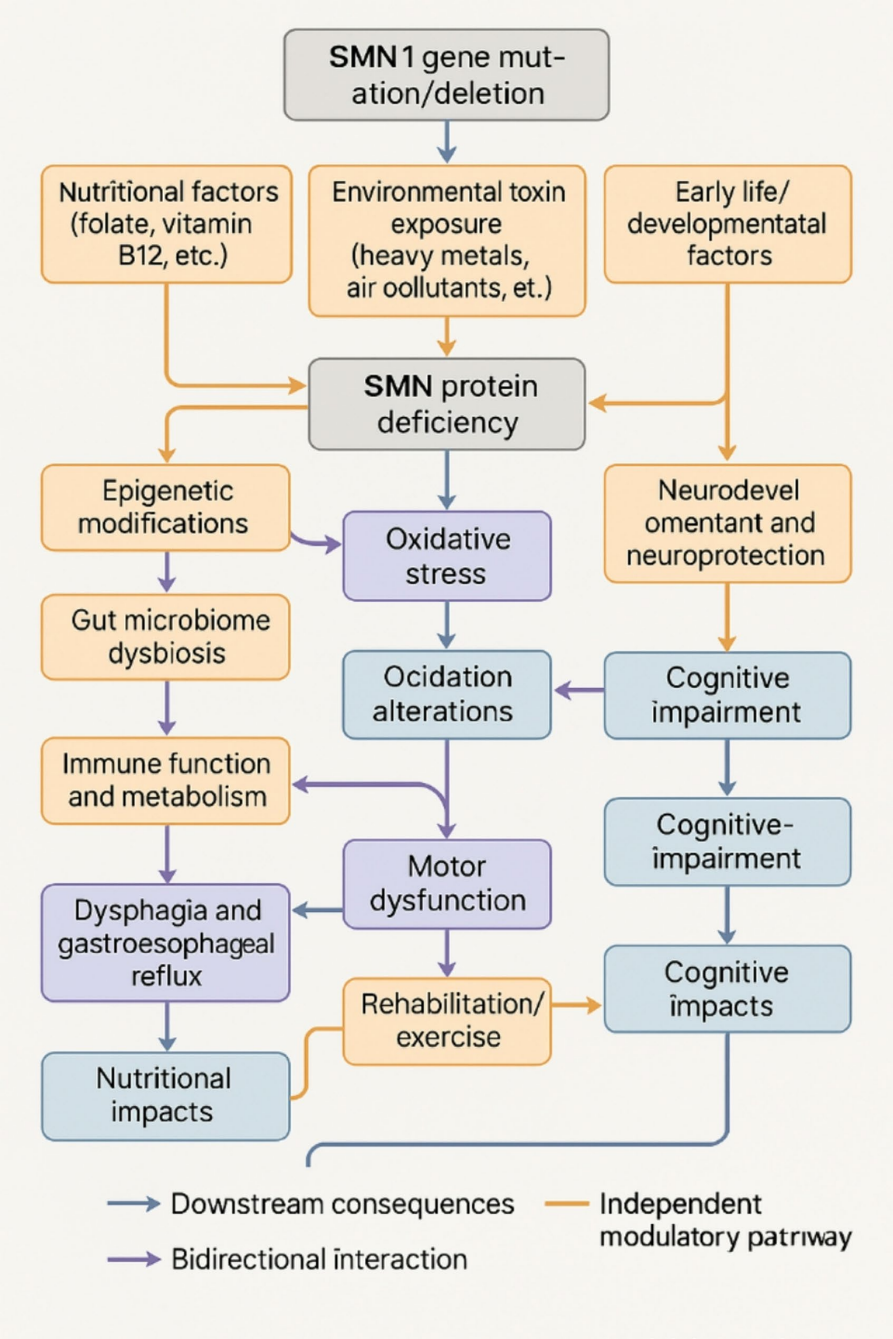
Bidirectional relationships between environmental factors and SMA disease progression Notes: Red arrows indicate downstream consequences of SMN deficiency, black arrows represent causal pathways that influence disease progression, and green arrows show bidirectional interactions. Environmental toxin exposure and early life factors can influence SMN gene expression through epigenetic modifications, while SMN deficiency leads to downstream effects including dysphagia, motor dysfunction, and metabolic alterations

Although it is mainly caused by *SMN1* gene defects, extensive research indicates that environmental factors play an important regulatory role in disease progression and phenotypic heterogeneity. These factors may alter the disease trajectories by affecting gene expression, protein function, or cellular metabolism. Figure 2 illustrates the bidirectional relationships between *SMN1* gene mutation/deletion as the primary cause and various environmental factors that can either result from SMN deficiency or independently modulate disease progression through distinct molecular pathways.

### Nutritional factors in SMA pathogenesis and management

Nutritional status has been widely recognized as an environmental factor influencing the health and quality of life of SMA patients. Nutritional perturbations in SMA exhibit a complex bidirectional relationship: on one hand, malnutrition and metabolic abnormalities predominantly represent downstream sequelae of muscular atrophy and neurological dysfunction resulting from SMN protein deficiency. On the other hand, specific nutrients may affect disease progression as independent regulatory factors. As research advances, targeted nutritional interventions predicated on this bidirectional relationship have emerged as an indispensable component of the comprehensive management strategy of SMA.

#### Nutritional challenges

SMA patients, particularly those with type 1 and type 2, universally experience complex nutritional challenges. These manifestations include growth restrictions, abnormal body composition, decreased bone density, and inadequate nutritional intake [50]. Clinical observations have identified dysphagia, gastroesophageal reflux, and gastrointestinal dysfunction as primary contributors to these nutritional complications, frequently precipitating malnutrition and growth retardation [51]. Malnutrition represents one of the foremost challenges confronting SMA patients, with approximately 60% of type 2 SMA children experiencing malnutrition, of which 35% meet criteria for severe malnutrition [52]. Further studies have shown that the risk of malnutrition in SMA patients is often underestimated, highlighting the imperative for comprehensive nutritional assessment and implementation of individualized nutritional management protocols [53, 54].

#### Specific nutrients

Studies have shown that specific nutrients may be involved in the SMA disease process, but their modes of action need to be distinguished. Metabolic abnormalities of certain nutrients, such as fatty acids, primarily represent downstream consequences of SMN protein deficiency, whereas other nutrients, such as folate and vitamin B12, may function as independent regulatory factors that modulate *SMN2* gene expression through epigenetic modifications, thereby affecting disease progression. Fatty acid metabolism dysfunction has been established as a core pathological feature of SMA, primarily resulting from SMN protein deficiency. Metabolomic analyses have revealed extensive fatty acid metabolic abnormalities in peripheral blood and spinal cord tissues of SMA animal models [55]. Based on this finding, researchers have proposed dietary interventions, such as Omega-3 fatty acid supplementation, to ameliorate SMA symptoms, which have been preliminarily validated in clinical [56]. This suggests that interventions targeting the downstream metabolic consequences of SMN deficiency may help improve patient prognosis. Multicenter studies of Chinese SMA children have further confirmed characteristic alterations in serum lipid profiles, emphasizing the necessity of incorporating lipid metabolism assessment into SMA nutritional management systems [57].

In terms of protein nutrition, research has demonstrated that the appropriate supply of protein and amino acids is crucial for maintaining muscle function in SMA patients. The SMAAF pilot study confirmed that specific amino acid formulations significantly improve motor function and developmental indicators of children with type 1 SMA [58]. In patients receiving disease-modifying treatments such as Nusinersen, simultaneous optimization of protein nutritional intake can substantially enhance therapeutic efficacy [59]. This clinical evidence has established important foundations for developing SMA-specific nutritional formulations. Furthermore, certain micronutrients also play a unique role in the pathological process of SMA. Folic acid and vitamin B12, as essential methyl donors, participate in epigenetic modifications such as DNA methylation and may influence the disease progression by regulation of *SMN2* gene expression. Clinical investigations have revealed widespread reductions in folate and vitamin B12 levels among type 1 SMA children, suggesting that supplementation of these nutrients may optimize *SMN2* expression and thus improve disease prognosis [60].

#### Gut microbiota

With advancing research into the gut-brain axis, the role of gut microbiota in neuromuscular diseases has garnered increasing attention. Studies indicate that gut dysbiosis may contribute to neuromuscular diseases by multiple pathways, including effects on immune function, energy metabolism, and neural development [61, 62]. Studies have shown that the intestinal microecology of patients with neuromuscular diseases is generally unbalanced, which may accelerate the progression of the disease [63]. Metabolomic analyses have demonstrated significant microbial metabolic abnormalities in SMA patients. A metabolomic study of cerebrospinal fluid from SMA patients identified marked alterations in multiple microbe-associated metabolites, suggesting that gut microecological dysregulation may participate in disease progression through modulation of host metabolism [64]. The changes in the intestinal flora observed in SMA patients primarily are mainly manifested as a decrease in the abundance of Firmicutes and an increase in the abundance of Proteobacteria. This change in flora structure is significantly correlated with lipid metabolism disorders in patients [65]. The role of gut microbiota in neuromuscular disorders has garnered substantial attention. Evidence indicates that intestinal microbial communities can influence the progression of neurodegenerative conditions through modulation of the neuroimmune axis [66]. Furthermore, there is evidence that the gut microbiome plays an important regulatory role in neurological diseases. Studies have shown that the gut microbiota mediate the anti-epileptic efficacy of ketogenic dietary interventions through the production of specific metabolites [67]. These findings suggest that gut microbiome-host metabolic interactions may contribute to the pathophysiological mechanisms and disease trajectory in SMA.

The results of studies conducted on animal models have further substantiated the intimate connection between gut microbiota and neuromuscular function [68, 69]. Research has found that gut dysbiosis not only affects the nervous system development but may also aggravate muscle dysfunction, including diaphragmatic impairment [70]. Prebiotic intervention has been shown to alleviate these symptoms to some extent, providing a new perspective for the development of therapeutic strategies based on microecological regulation [71, 72]. Consequently, researchers have proposed therapeutic strategies aimed at improving SMA patient outcomes through the modulation of gut microbiota, encompassing nutritional interventions such as dietary fiber, prebiotics, and probiotics [73]. These interventions show promise for optimizing gut microbiota composition and improving patients’ metabolic status and neuromuscular function [74]. However, more clinical studies are required to evaluate its effectiveness and safety.

### Early life experiences and SMA prognosis

As a progressive neuromuscular disease, the prognosis and quality of life of SMA are influenced by multiple factors. Research indicates that early postnatal experiences, including therapeutic interventions, rehabilitation management, and psychosocial support, are crucial to the long-term prognosis of patients.

#### Early diagnosis and therapeutic intervention

With the advancement of newborn screening technology and the improvement of clinical diagnosis and treatment, the possibility of early SMA identification and intervention has increased significantly. Clinical studies demonstrate that patients who start treatment during the pre-symptomatic phase achieve superior motor function developmental outcomes compared to those beginning treatment post-symptom onset [75, 76]. Furthermore, early diagnosis and treatment can delay disease progression and improve survival quality [77]. Regarding therapeutic approaches, SMN-dependent treatments represented by Nusinersen, Zolgensma, and Risdiplam have emerged as core strategies for early intervention, and demonstrated significant therapeutic efficacy in clinical applications [78]. However, monotherapy may prove insufficient to completely halt disease progression in severe SMA patients.

#### Rehabilitation therapy

Rehabilitation therapy, recognized as a crucial component of comprehensive SMA management, has garnered widespread acknowledgment. The early implementation of individualized rehabilitation programs not only helps maintain the patient’s muscle strength and motor function, but also effectively prevents complications and improves the quality of life [79, 80]. International rehabilitation guidelines emphasize the necessity of continuous intervention while noting significant variations in rehabilitation training tolerance and response among different SMA types [81]. Particularly for patients with severe diseases such as type 1, precise control of exercise intensity is essential to avoid muscle injury and fatigue from overtraining [82]. Therefore, rehabilitation programs need to be tailored to the individual characteristics and functional status of the patient [83]. In addition, innovative rehabilitation models involving multidisciplinary collaboration can provide more comprehensive and continuous rehabilitation services for SMA children, potentially further improving their motor, cognitive, and social adaptation abilities [84].

#### Psychosocial support and quality of life

SMA, as a complex neuromuscular disorder, extends far beyond physical dysfunction, profoundly impacting patients’ psychological health and social participation. Research indicates that the development of self-transcendence capabilities and social support networks represents key factors in enhancing the quality of life of SMA patients [85]. Systematic interview studies have further revealed multiple challenges faced by SMA families, including diagnostic uncertainty, overwhelming daily care burdens, and social prejudice [86]. Longitudinal studies conducted during clinical trials have provided important insights. For instance, parental interview data from Nusinersen clinical trials demonstrate that standardized medical interventions not only improved the clinical condition of children, but also significantly enhanced families’ disease coping abilities and self-efficacy through established support networks and peer communication platforms [87]. The positive role of this psychosocial support emphasizes the importance of holistic care in SMA management. Multinational multicenter studies have provided crucial basis for developing comprehensive management strategies. Quality of life assessments based on cross-survey methodologies indicate that early multidisciplinary intervention significantly improves patient prognosis and reduces family burden [88]. European-wide health economics analyses further confirm that timely diagnosis, standardized treatment, and continuous rehabilitation support not only improve health-related quality of life but may also reduce long-term medical costs [89].

#### Early experience effects on SMA epigenetic regulation

Early life experiences profoundly influence SMA disease progression through epigenetic mechanisms. Research has revealed two primary regulatory pathways, neuroprotective and neurodegenerative. In terms of neuroprotection, moderate exercise stimulation can induce upregulation of crucial genes such as brain-derived neurotrophic factor. This epigenetic change helps maintain the survival of motor neurons and synaptic plasticity. Conversely, early stress exposure affects the function of the hypothalamic-pituitary-adrenal axis through epigenetic modifications, leading to neuroendocrine system dysregulation and accelerated neurodegenerative processes [90]. Molecular mechanism studies have shown that SMN protein deficiency not only affects motor function but may also impair cognitive development through disruption of epigenetic modification processes during neurodevelopment [91]. Notably, enriched sensorimotor stimulation and positive family interactions may exert neuroprotective effects through inducing beneficial epigenetic changes. However, systematic studies examining how early experiences shape the epigenetic landscape in SMA patients remain lacking.

### Potential impact of environmental pollutants and chemical exposures on SMA

Although direct evidence linking environmental pollutants to SMA remains limited, research suggests these factors may potentially influence disease progression. Environmental agents such as heavy metals, endocrine disruptors, air pollutants, and pesticides may affect neuronal health through mechanisms including oxidative stress induction and epigenetic modifications.

#### Heavy metal exposure and SMA

Heavy metals exhibit neurotoxicity through mechanisms including oxidative stress and mitochondrial dysfunction [92]. Recent clinical studies found that serum copper levels in SMA patients are significantly higher than in healthy controls (1.28 ± 0.31 mg/L vs. 1.02 ± 0.28 mg/L, *P* < 0.001), with copper levels positively correlating with body fat percentage (*r* = 0.42, *P* < 0.01) [93]. Similar abnormalities have been observed in other motor neuron diseases [94–96]. However, definitive evidence for a causal relationship between heavy metal exposure and SMA pathogenesis remains lacking [97], and large-scale clinical studies have failed to confirm associations between heavy metal levels and disease phenotypes [98, 99].

#### Neurotoxicity of endocrine disruptors

Endocrine disruptors (EDCs) interfere with hormone systems and include substances such as bisphenol A, PFAS, and flame retardants. EDCs can affect nervous system development and function through multiple mechanisms [100, 101]. Bisphenol A affects neuronal development at concentrations of 10–100 nM [102], while PFAS exposure can induce oxidative stress and mitochondrial dysfunction at environmentally relevant levels [103]. Organophosphate flame retardants can cause neurotoxicity below regulatory standards [104], partly by affecting myelination [105]. Prenatal EDC exposure may “pre-program” neurological susceptibility [106]. However, direct evidence for EDCs in SMA pathogenesis remains limited.

#### Neurotoxicity of air pollutants

Air pollutants show associations with neurological damage and neurodegenerative disease risk through mechanisms including oxidative stress, inflammatory responses, blood-brain barrier disruption, and microglial activation [107]. PM2.5 exposure demonstrates dose-dependent effects, increasing oxidative stress markers and decreasing myelin protein expression [108]. Inflammatory factors increase neurodegenerative disease risk by 15–20% for every 10 µg/m³ increase in PM2.5 [109]. While direct evidence linking air pollutants to SMA is lacking, these agents might potentially influence disease progression through inflammation and oxidative mechanisms.

#### Pesticide exposure and motor neuron disease

Epidemiological studies link occupational pesticide exposure to increased risk of motor neuron diseases [110, 111]. Organochlorine, organophosphorus, and carbamate compounds show selective toxicity to motor neurons through cholinesterase inhibition, oxidative stress, mitochondrial dysfunction, and axonal transport disruption [111]. Organophosphate pesticides reduce motor neuron survival by approximately 40% at concentrations of 0.1-1.0 µM, while chronic low-dose exposure can alter gene expression through epigenetic mechanisms [110]. The potential relevance of these findings to SMA warrants further investigation.

### Interaction between environmental factors and epigenetics

A complex bidirectional regulatory network exists between environmental factors and epigenetic mechanisms, influencing *SMN2* gene expression and SMA pathological processes through multiple molecular pathways. In the molecular pathophysiology of SMA, *SMN1* gene deletion or mutation constitutes the primary etiological determinant, while environmental factors and epigenetic modifications serve as disease modifiers rather than the primary causative agents. Specifically, environmental factors may influence SMA through three distinct pathways: (1) as downstream sequelae of SMN deficiency, exemplified by nutritional intake difficulties resulting from motor dysfunction; (2) as disease modifiers that regulate *SMN2* gene expression through alterations in epigenetic modifications; and (3) as independent pathological factors that directly accelerate motor neuron degeneration through mechanisms such as oxidative stress. Among these pathways, environmental factors regulating *SMN2* gene expression through epigenetic modifications represents the most promising target for therapeutic intervention. Research demonstrates that environmental factors affect epigenetic modifications through several key pathways. Nutritional status regulates DNA methylation through one-carbon metabolism pathways, where folate and vitamin B12 serve as methyl donors, influencing DNA methylation patterns through S-adenosylmethionine (SAM)-dependent methyltransferase activity. Omega-3 fatty acids regulate the activity of histone deacetylase by activating the *SIRT1/AMPK* pathway, thereby changing the chromatin state. Furthermore, environmental toxin exposure can influence epigenetic regulatory factor expression and activity through oxidative stress-induced activation of *NF-κB* and *MAPK* signaling pathways [17, 55].

Environmental factors also influence epigenetic modifications through cellular metabolic pathways. Nutritional stress modulates histone-modifying enzyme activity via the *mTOR* signaling pathway, while oxidative stress affects the function of cofactor-dependent histone modifying enzymes through alterations in NAD+/NADH ratios. These metabolic changes ultimately influence gene expression through regulation of histone acetylation and methylation levels. Concurrently, epigenetic states determine cellular response patterns to environmental stimuli [18]. Alterations in DNA methylation levels can affect the promoter activity of stress response genes, thereby regulating cellular sensitivity to environmental stress, while histone modification states modulate stress response gene expression through influencing transcription factor accessibility. In SMA patients, hypermethylation of the *SMN2* gene promoter region may increase susceptibility to environmental stress, while epigenetic alterations in genes related to mitochondrial function may exacerbate sensitivity to malnutrition and oxidative stress [55]. Noncoding RNA networks also play an important role in the interaction between environmental factors and epigenetic regulation. Environmental stress can induce the expression of specific miRNAs, which influence gene expression by targeting epigenetic regulatory factors. Simultaneously, epigenetic modifications can regulate miRNA transcription, forming complex feedback regulatory circuits.

A deeper understanding of the interactions of these molecular pathways will help elucidate the role of environmental factors in SMA pathogenesis and provide new perspectives for developing targeted therapeutic strategies. However, the current knowledge of these complete regulatory networks and their SMA-specific functions remains limited, necessitating additional mechanistic studies. Future research should focus on the spatiotemporal specificity of epigenetic changes induced by environmental factors, the synergistic effects between different epigenetic modifications, the dose-response relationship between environmental factors and epigenetic regulation, and the development of individualized therapeutic strategies. These investigations will contribute to a more comprehensive understanding of SMA pathological mechanisms, providing scientific foundations for optimizing prevention and treatment strategies.

## Therapeutic implications of epigenetic and environmental factor research in SMA

Epigenetic and environmental factor research has not only deepened our understanding of SMA pathogenesis but also provided crucial insights for developing novel therapeutic strategies and optimizing existing treatments. Through drug intervention targeting epigenetic modification processes and controlling exposure to environmental risk factors, it is expected that precise treatment and personalized management of SMA can be achieved.

### Potential of epigenetic targeted therapy

#### DNA methylation modulators

DNA methylation represents one of the most extensively studied epigenetic modifications in SMA. Research has revealed widespread DNA methylation abnormalities in motor neurons and skeletal muscle of ALS and SMA patients and *SOD1* mouse models, with targeted DNA methylation intervention demonstrating phenotypic improvements [19]. Further studies have shown the therapeutic potential of specific DNA methylation modulators in SMA treatment [33]. Additionally, DNA methylation may mediate the neuroprotective effects of environmental interventions such as exercise training and β2-adrenergic receptor agonists [112].

#### Histone deacetylase inhibitors

Histone acetylation plays a crucial role in the pathogenesis of SMA. HDAC inhibitors such as trimebutine and valproic acid have shown the potential to upregulate *SMN2* gene expression [16, 38, 113]. Furthermore, HDAC inhibitors can also exert therapeutic effects independent of SMN by improving metabolism and reducing muscle atrophy [114, 115]. The development of specific HDAC inhibitors and the optimization of dosing regimens are expected to further improve their efficacy and safety [116, 117].

#### Noncoding RNA therapeutics

Targeting aberrantly expressed noncoding RNAs provides novel therapeutic approaches for SMA. Antisense oligonucleotides (ASOs) can specifically silence the *SMN2* antisense transcript *SMN-AS1*, thereby upregulating SMN2 expression [14]. miRNA replacement therapy and inhibitors have also demonstrated potential in ameliorating SMA phenotypes [118, 119]. Additionally, the detection of miRNA profiles in the cerebrospinal fluid and blood of SMA patients holds promise for developing novel disease biomarkers and therapeutic monitoring indicators [120–122].

#### Chromatin remodeling modulators

Chromatin remodeling complexes such as SWI/SNF play pivotal roles in regulating *SMN2* gene transcription. The development of specific chromatin remodeling modulators shows promise for enhancing *SMN2* expression levels [123, 124]. Additionally, RNA-binding proteins such as Sam68 and CARM1 are involved in the pathogenesis of SMA by affecting the circularization and splicing of *SMN2* pre-mRNA, and may also become potential therapeutic targets [125–127].

### Potential of environmental intervention

Despite the paucity of direct evidence regarding the efficacy of adjunctive interventions in SMA treatment, emerging research suggests that optimized nutritional support and rehabilitation protocols may ameliorate patient outcomes. SMA patients frequently experience substantial challenges related to malnutrition and metabolic dysregulation. Existing studies have provided basic nutritional assessment data, but personalized nutritional programs for different types of SMA patients still need to be further explored [52, 53]. Rehabilitation therapy constitutes an indispensable component of comprehensive SMA management. Moderate physical activity not only preserves muscular functionality but may also attenuate disease progression through the induction of neuroprotective epigenetic modifications. Evidence indicates that the concomitant administration of myostatin inhibition and antisense oligonucleotide therapy yields enhanced prognostic outcomes in SMA, suggesting that the formulation of individualized exercise protocols in conjunction with SMN dependent therapeutic modalities may optimize clinical efficacy [128]. Subsequent research endeavors should prioritize the evaluation of potential synergistic interactions between these adjunctive interventions and pharmacological approaches, as well as elucidating the underlying mechanisms and scientific basis for their therapeutic efficacy.

### Precision medicine and individualized therapy

**Fig. 3:**
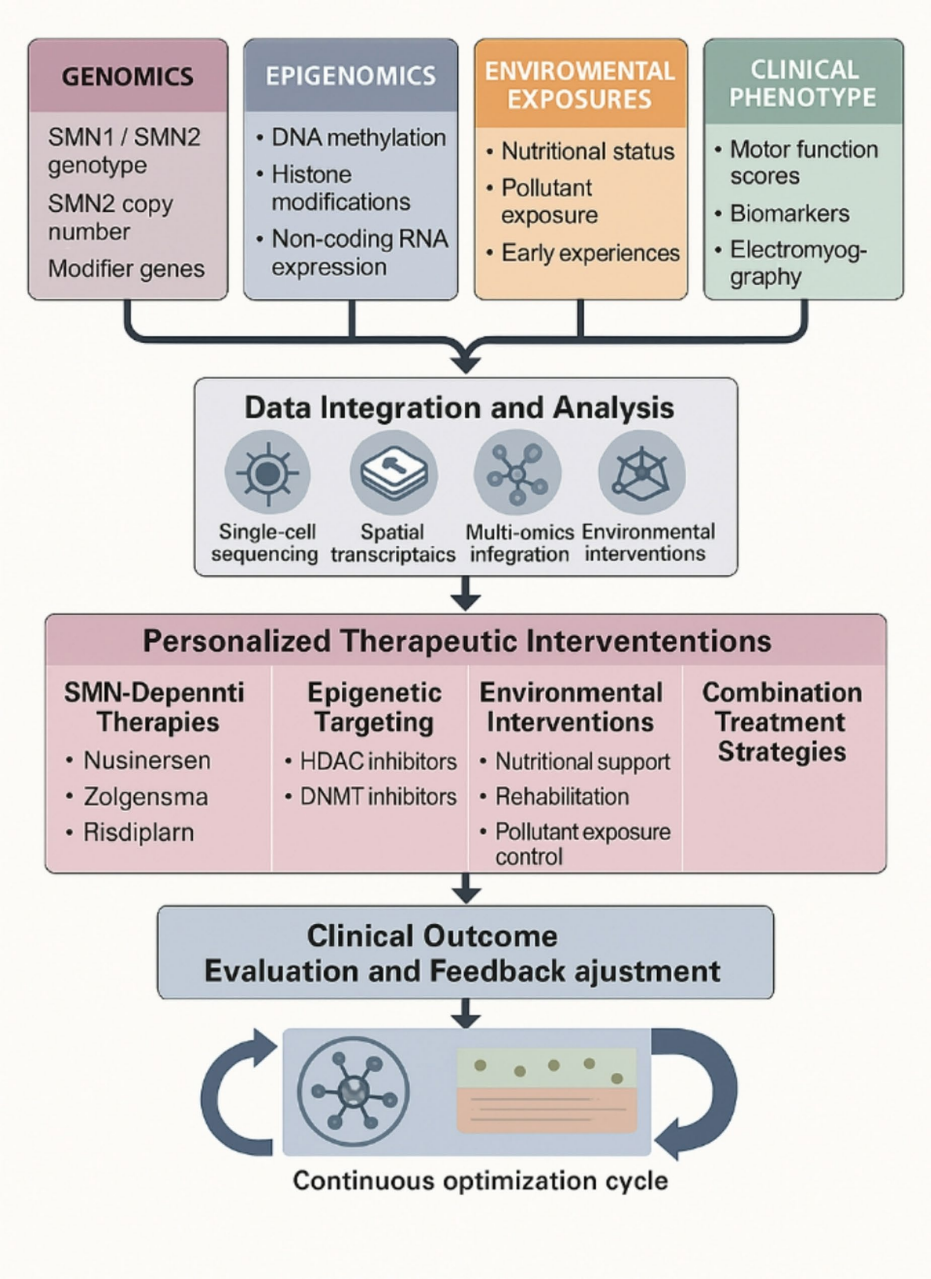
Integrative model for personalized precision medicine in SMA Notes: This model illustrates the integration of multiple data streams (genomic, epigenomic, environmental, and clinical) through advanced analytical methods to inform personalized therapeutic strategies. The approach combines SMN-dependent therapies, epigenetic-targeted interventions, and environmental modifications based on individual patient profiles

Epigenetic and environmental factor research has established foundations for precision medicine in SMA. Systematically integrating genomic, epigenomic, metabolomic, and environmental exposure data makes it feasible to develop personalized disease risk prediction models and biomarker profiles [9]. Although the approval and implementation of SMN-dependent therapies have significantly improved SMA patient prognosis, substantial clinical heterogeneity persists. This suggests the necessity of transitioning from a “one-size-fits-all” treatment model toward individualized precision intervention strategies (Fig. 3) to further enhance therapeutic efficacy and quality of life [129]. In formulating therapeutic decisions, consideration must be given to patient-specific epigenetic backgrounds, environmental exposure histories, and responsiveness to approved treatment regimens to achieve optimal balance between efficacy and safety. Furthermore, combining epigenetic pharmaceuticals with SMN-dependent therapies shows promise for producing synergistic effects [130]. Future SMA treatments should focus on the combined use of multiple treatments, including SMN replacement therapy, SMN-independent therapy, and peripheral treatment strategies based on the interaction mechanism between the central nervous system and the peripheral system [129].

## Research challenges and future directions

Despite significant advances in SMA epigenetic and environmental factor research, numerous challenges persist. Current studies predominantly focus on analyzing single epigenetic modification types or environmental factors, lacking systematic understanding of dynamic multi-factor interactions in SMA pathology. Additionally, research primarily concentrates on peripheral tissues, with limited understanding of the epigenetic foundations of central nervous system pathology. Sample size limitations, insufficient longitudinal follow-up data, and disease model constraints have hindered translational applications. The development of new technologies provides an opportunity to overcome these challenges. The application of single-cell sequencing technology (scRNA-seq) has revealed the heterogeneity of spinal motor neurons, providing new perspectives on SMA pathological mechanisms [131]. Spatial transcriptomics technology preserves tissue spatial information, facilitating characterization of molecular features in pathological regions [132]. The single-cell transcriptome map of human spinal cord development provides an important reference for studying the developmental lesions of SMA [133]. However, the application of these new technologies also faces challenges in data analysis, sample processing and result interpretation, and standardized experimental and analytical processes need to be established. Furthermore, early transcriptome studies of patients treated with Nusinersen demonstrated the value of multi-omics analysis in therapeutic efficacy assessment [134]. However, the application of these new technologies also faces challenges in data analysis, sample processing and result interpretation, and standardized experimental and analytical processes need to be established.

A pivotal direction for future investigation lies in establishing a definitive delineation between the direct sequelae of SMN deficiency and independent disease-modulating pathways. Current evidence suggests that nutritional metabolic dysregulation and muscular atrophy predominantly represent direct consequences of SMN protein insufficiency, whereas specific environmental exposures such as certain chemical compounds and epigenetic modifications may affect disease progression as independent regulatory pathways. The implementation of conditional gene knockout animal models coupled with precise molecular pathway analyses will facilitate more accurate characterization of these factors within the pathological continuum of SMA, thereby establishing a theoretical framework for the development of increasingly targeted therapeutic interventions.

Future research should adopt integrated multi-omics analysis strategies in larger cohorts. The utilization of dynamic monitoring techniques, encompassing the domains of epigenomics, transcriptomics, proteomics and metabolomics, in conjunction with advanced single-cell technologies and spatial transcriptomics, facilitates a precise analysis of the pathological alterations within the central nervous system of patients diagnosed with SMA. This analysis unveils the presence of spatial heterogeneity in the progression of the disease. Molecular analysis of central nervous system specimens, such as cerebrospinal fluid and brain tissue, will facilitate a more profound comprehension of the disease mechanism. These analyses can be combined with humanized disease models, such as the establishment of SMA-specific neuronal and glial cell models through iPSC technology, and the in-depth exploration of the molecular mechanisms of epigenetic modification and environmental factors. The integration of clinical phenotypes, environmental exposure information, and multi-omics data facilitates the construction of a more accurate disease prediction model, providing a foundation for the development of individualized treatment plans.

An emerging research direction that requires attention is the role of gene-environment interactions (GEI) in SMA. Although *SMN1* gene deletion is the primary cause of SMA, the heterogeneity in clinical phenotypes suggests complex interactions between genetic background and environmental factors. Recent studies in other neurodegenerative and neurodevelopmental disorders have shown that environmental exposures can significantly influence disease phenotypes and progression through epigenetic mechanisms [135–137]. For example, in Alzheimer’s disease, interactions between APOE genotype and air pollutant exposure can significantly affect the rate of cognitive decline [137]. In Parkinson’s disease, interactions between certain pesticide exposures and specific genetic polymorphisms can increase disease risk [136]. These findings provide important insights for understanding the heterogeneity of SMA. Future SMA research should adopt prospective cohort designs, collect detailed environmental exposure data, and combine genetic analyses with epigenomic profiling to systematically evaluate how gene-environment interactions regulate *SMN2* gene expression and influence disease phenotypes [138]. Recent advances in computational methods, including network-based approaches and machine learning algorithms, can help identify key gene-environment interaction networks from complex multi-omics data, as demonstrated in amyotrophic lateral sclerosis research where multiomics integration with artificial intelligence successfully uncovered novel transcriptional and mutational signatures and previously uncharacterized disease pathways, suggesting similar computational strategies could be applied to discover complex gene-environment interactions in SMA [139]. Additionally, patient-derived induced pluripotent stem cells (iPSCs) have proven valuable for modeling SMA pathophysiology and can be used to study the effects of specific environmental exposures on motor neurons with different genetic backgrounds in controlled environments [140]. These studies will not only deepen our understanding of SMA pathogenesis but also provide a basis for developing individualized prevention and treatment strategies, such as early intervention measures targeting specific modifiable factors, or epigenetic-targeted therapies tailored to patients’ genotypes [141].

## Conclusion

This review summarizes the important progress of epigenetic research in clarifying the pathogenesis of SMA and developing new treatment strategies, while also noting how epigenetic mechanisms may interface with other modulatory factors. Epigenetic modifications, including DNA methylation, histone modifications, and non-coding RNAs, influence SMA phenotypes by regulating the expression and splicing of the *SMN2* gene. Simultaneously, environmental factors such as nutrition, pollutant exposure, exercise, and stress can also affect *SMN2* gene expression and SMA pathological processes through epigenetic pathways. Despite the present limitations of current studies, these constraints may be gradually overcome through application of multi-omics integration analysis and advanced models. Future research should strengthen multidisciplinary collaboration, integrate genetic, epigenetic, and environmental factor data, construct individualized risk prediction and therapeutic efficacy assessment models, and accelerate translation of research findings into clinical applications.

## Data Availability

Data sharing is not applicable to this article as no datasets were generated or analyzed during the current study.
